# Xingnao Jieyu Decoction Ameliorates Poststroke Depression through the BDNF/ERK/CREB Pathway in Rats

**DOI:** 10.1155/2018/5403045

**Published:** 2018-10-16

**Authors:** Tao Li, Dou Wang, Bingbing Zhao, Yongmei Yan

**Affiliations:** ^1^Department of Encephalopathy, Affiliated Hospital of Shaanxi University of Chinese Medicine, West Weiyang Road, Xianyang, Shaanxi 712000, China; ^2^First Clinical Medical College, Shaanxi University of Chinese Medicine, West Weiyang Road, Xianyang, Shaanxi 712000, China

## Abstract

*Background. *The neurotrophic pathway regulated by the brain-derived neurotrophic factor (BDNF) plays a crucial role in the pathogenesis of poststroke depression (PSD). How the traditional Chinese medicine compound preparation Xingnao Jieyu (XNJY) decoction regulates the neurotrophic pathway to treat PSD is unclear.* Objective.* This study aimed to investigate the antidepressant effect of XNJY decoction on a rat model of PSD and the molecular mechanism intervening in the neurotrophic pathway.* Methods. *After a middle cerebral artery occlusion model was established, chronic unpredictable mild stress was applied for 21 days to prepare a PSD model. XNJY groups and a fluoxetine (Flu) group of rats were intragastrically administered with XNJY and Flu, respectively, for 21 consecutive days. Depressive-like behaviors, including sucrose preference, open field test, and forced swimming test, were assessed. The survival and apoptosis of cortical and hippocampal neurons were evaluated by immunofluorescence assay and TUNEL staining. The contents of serotonin (5-HT), norepinephrine (NE), and BDNF in the cortex and hippocampus were determined by ELISA. The protein levels of BDNF, p-ERK/ERK, and p-CREB/CREB in the cortical and hippocampal regions were tested by Western blot.* Results. *The depressive-like behaviors markedly improved after XNJY and Flu treatment. XNJY and Flu promoted neuronal survival and protected cortical and hippocampal neurons from apoptosis. XNJY also increased the contents of 5-HT, NE, and BDNF and recovered the protein levels of p-ERK/ERK, p-CREB/CREB, and BDNF in the cortical and hippocampal regions.* Conclusion.* These results indicated that the XNJY decoction exerts an obvious antidepressant effect, which may be due to the regulation of the BDNF/ERK/CREB signaling pathway.

## 1. Introduction

Poststroke depression (PSD) affects one-third of stroke survivors and is the most common emotional disorder after stroke [1, 2]. This condition negatively affects the ability of patients with stroke to engage in rehabilitation and is strongly associated with the recovery of physical, cognitive, and functional outcomes and quality of life [3]. Unfortunately, these mood and emotional disturbances are not apparent and often ignored by clinicians [4]. With the increasing incidence of PSD following the growth of the aged population and occurrence of stroke at a young age, the prevention and treatment of PSD are becoming increasingly important and must be urgently realized. Currently, the treatment for PSD is mainly dependent on antidepressant drugs, including tricyclic antidepressants and selective serotonin (5-HT) reuptake inhibitors. However, tricyclic antidepressants were reported to have adverse effects in the liver, kidney, and heart. Selective serotonin reuptake inhibitors were associated with increased risk for stroke, intracranial hemorrhage, and all-cause mortality, so conventional antidepressants are limited in their clinical application [5–7].

Multiple pathogenetic mechanisms exist in PSD and include imbalance of monoamine transmitters, excessive neuroinflammation, dysfunction of the hypothalamic-pituitary-adrenal axis, enhanced glutamate-mediated excitotoxicity, and deficiency of neurotrophins [8–10]. Among these mechanisms, the neurotrophic hypothesis plays crucial roles in the pathophysiology of PSD by affecting neuronal survival, development, and plasticity [11]. The brain-derived neurotrophic factor (BDNF), as a regulator of neuronal survival and neurogenesis, is believed to be involved in the protection and recovery of functions after stroke [12, 13]. Strong evidence has revealed a reduction in mRNA and protein levels of BDNF in patients and rat model of depression relative to those of controls and recovery after antidepressant treatment [14–17]. Besides, the activation of BDNF can regulate downstream molecules, such as ERK and cAMP response element-binding (CREB) protein. These transcription factors also induce the secretion of neurotrophic factors and promote neuronal survival, repair, and regeneration [18]. Thus, the neurotrophic pathway regulated by BDNF may be a potential target for PSD therapeutic intervention.

Many traditional Chinese medicine (TCM) prescriptions display antidepressant effects with few side effects and multiple targets and possess great potential in treating PSD [19]. Xingnao Jieyu (XNJY) decoction, as a TCM compound preparation, has been shown to alleviate depressive systems and exhibit minimal side effects in patients with PSD by eliminating phlegm, dissolving blood stasis, restoring consciousness, and relieving depression. Our previous studies revealed that XNJY exerts antidepressant effects in rats with PSD by upregulating synaptotagmin expression in the hippocampus and improving neuronal morphology in the frontal lobe and hippocampus [20, 21]. To further clarify the influence of XNJY on the neurotrophic pathway, we established the rat model of PSD by using middle cerebral artery occlusions (MCAO) in combination with chronic unpredictable mild stress (CUMS). We then evaluated the effects of XNJY on neuronal survival and apoptosis in the hippocampus and cortex and tested the influence of XNJY on the cortical and hippocampal contents of 5-HT, norepinephrine (NE), and BDNF, as well as the BDNF/ERK/CREB signaling pathway, in the PSD rat model.

## 2. Materials and Methods

### 2.1. Materials

XNJY decoction comprises Acorusgramineus (Shichangpu, 12 g), Radix Polygalae (Yuanzhi, 12 g), Radix Salviae Miltiorrhizae (Danshen, 30 g), Radix Bupleuri Chinensis (Chaihu, 12 g), Silktree Albizzia bark (Hehuanpi, 15 g), Radix Curcumae (Yujin, 10 g), and Radix Morindae Officinalis (Bajitian, 10 g). All Chinese herbal pieces were provided by the Preparation Center in the Affiliated Hospital of Shaanxi University of Chinese Medicine in accordance with the standards listed in the National Pharmacopeia of China. The XNJY decoction was prepared by boiling the mixture in distilled water at 100°C for 30 min twice. The drug solution was condensed on the basis of two XNJY treatment groups (XNJY-10.5g/kg and XNJY-21g/kg), and the concentrated solution was intragastrically administered at 3 mL per rat. Flu was obtained from Lilly (#J20030017, Suzhou, China). A TUNEL assay kit was purchased from Roche (#11684817910, USA). Rat 5-HT (SBJ-R0025), NE (SBJ-R0128), and BDNF (SBJ-R0021) ELISA kits were obtained from Senbeijia Biotechnology (China). Antibodies, including anti-p-ERK1/2(#4695), anti-ERK1/2 (#4696), anti-CREB (#9104), and anti-p-CREB (#9198), were purchased from Cell Signaling Technology (USA). Anti-BDNF (1:2000, ab108329) was obtained from Abcam (USA). Anti-Neuron (GB11138-1) and CY3-conjugated goat anti-rabbit IgG (GB21303) were obtained from Servicebio (China). HRP-conjugated goat anti-rabbit IgG (1:5000, MF094) and HRP-conjugated goat anti-mice IgG (1:5000, MF093) were purchased from Mei5 (China).

### 2.2. Animals and Administration Procedure

Sprague–Dawley rats (72 rats, male, weighing 180 ± 20 g) were supplied by the Medical Experimental Animal Center of the Xi'an Jiaotong University (Xi'an, China). The procedure protocol was approved by the Animal Care and Use Committee of the Xi'an Jiaotong University. The rats were maintained under standard laboratory conditions (22 ± 2°C, 50% ± 10% humidity, and a 12 h/12 h light/dark cycle) with food and water* ad libitum* for 1 week of adaptation.

After MCAO or Sham-operated treatment, the rats were kept in cages without any stress and given free access to food and water for 2 days. Neurological deficit scoring was performed, and the stroke rats were randomly divided into six groups of 12 rats each. The Sham group included Sham-operated rats intragastrically administered with saline for 21 days. The MCAO group comprised MCAO rat models intragastrically administered with saline for 21 days. The PSD group included MCAO rat models treated with unpredictable mild stress for 21 days. The Flu group comprised MCAO rat models treated with unpredictable mild stress and intragastrically administered with Flu (2.08 mg/kg) for 21 consecutive days. The XNJY groups involved MCAO rat models treated with unpredictable mild stress and intragastrically administered for 21 consecutive days with different doses of XNJY decoction (XNJY-10.5 g/kg and XNJY-21g/kg, respectively, equivalent to the dry weight of raw materials).

### 2.3. Neurological Deficit Score

The neurological deficit score was applied using a five-point scale system. A score of 0 indicated no neurologic deficit, a score of 1 (failure to extend left forepaw fully) indicated a mild focal neurologic deficit, a score of 2 (circling to the left) indicated a moderate focal neurologic deficit, a score of 3 (falling to the left) indicated a severe focal deficit, and a score of 4 (no spontaneous locomotor activity or barrel rolling) indicated a critical focal neurologic deficit. The rats with no significant neurologic deficit were removed from further study.

### 2.4. Stroke and PSD Model

The stroke rat model was prepared through MCAO [22]. After MCAO surgery, all rats were kept in cages without any stress and given free access to food and water for 2 days. The neurological deficit score was determined through the Zea-Longa5 method, and the stroke rats were subjected to CUMS for 21 consecutive days to establish the PSD model on the basis of a modified Willner's method [23]. The CUMS included food and water deprivation (24 h), shaking (200 Hz for 5 min), inversion of light/dark cycle (24 h), tail nipping (2 min), soiled cage (100 mL of water spilled onto the bedding, 24 h), forced swimming in cold water (4°C) for 5 min, and electric shock (0.9 mA, 15 s × 4 times). Each stressor method was used randomly three times during the CUMS procedure.

### 2.5. Body Weight Assessment and Sucrose Preference Test

The body weight and sucrose water consumption of rats in each group were assessed on the day before MCAO and on the day after the last CUMS. The sucrose preference test was performed by providing one bottle of 1% sucrose water and one bottle of standard drinking water after 24 h of water and food deprivation. The consumption of sucrose water and standard water in 1 h was calculated, and sucrose preference was evaluated in accordance with the following formula: sucrose intake/(sucrose intake + water intake) × 100%.

### 2.6. Open Field Test

The open field test was performed using an open box (80 cm × 80 cm × 50 cm) to investigate locomotor activity after the last CUMS. The box floor was divided into 25 equal squares, and the rats were quickly placed at the center of the box individually. After a 5 min test, the line crossings (number of times that the rats crossed one of the grid lines with all four paws) were counted as horizontal score. The frequency of two forelimbs leaving the ground was calculated as the vertical score.

### 2.7. Forced Swimming Test

The forced swimming test was used to evaluate depression-like behavior after the last CUMS. The rats were forced to swim individually in a glass cylinder (15 cm in height and 10 cm in diameter) filled with water (25 ± 1°C). All the animals were forced to swim for 5 min, and the immobility time (including passive swimming) during the test was recorded by two blinded observers.

### 2.8. TUNEL Staining

TUNEL assay was used to detect apoptosis in accordance with the manufacturer's protocol (Roche, USA). The rats were anesthetized with chloral hydrate (350 mg/kg, i.p.) and perfused with cold heparinized saline after treatment. The brains were removed, fixed in 4% formaldehyde in PBS for 24 h, and dehydrated with different concentrations of ethanol. The samples were then embedded in paraffin for sectioning. The brains were cut into 5 *μ*m-thick coronal sections and stained with the TUNEL assay kit. The slides were observed under optical microscopy (DP73, Olympus). Apoptotic neurons in the ischemic cortex and hippocampus were counted for five fields per section by a blinded observer.

### 2.9. ELISA Assay

Neurotransmitters, such as 5-HT, NE, and BDNF, were detected by ELISA. The hippocampus and cortex were isolated from the rat brains and homogenized in PBS on ice for 30 min. After centrifugation at 13,000 rpm, we collected the supernatant into a centrifuge tube. We then added the standard dilution and samples to the enzyme-coated plates successively in accordance with the protocol. After incubation for 30 min, the liquid in the plate was discarded, and the plate was washed with washing buffer. After adding reagents A and B to the plate and evading light preservation for 15 min at 37°C, we stopped the reaction by adding termination solution to each well and tested the value at 450 nm absorbance by a microplate reader (Bio-Rad, Hercules, USA) within 15 min.

### 2.10. Immunofluorescence Assay

Immunofluorescence assay was performed to investigate the surviving neurons. Brain slices were permeabilized with 0.3% Triton X-100 for 1 h and incubated with 1% bovine serum albumin for 1 h at room temperature. After being washed with PBS three times, the slices were incubated with anti-neuron antibody (1:500) at 4°C overnight. The next day, the slices were washed with PBS and labeled with CY3-conjugated goat anti-rabbit IgG (1:200) for 2 h at room temperature while avoiding light. After DAPI staining, images were acquired using a Pannoramic MIDI digital slide scanner (3D HISTECH, Hungary). The NeuN-positive cells were counted in three random images from each brain section by a technician blinded to this study.

### 2.11. Western Blot Assay

Rats were anesthetized and perfused with cold heparinized saline after treatment. The cortex and hippocampus were isolated from the rat brains and homogenized in lysis buffer (50 mM Tris–Cl, 150 mM NaCl, 0.02% NaN_2_, 100 *μ*g/mL phenylmethanesulfonyl fluoride, 1 *μ*g/mL aprotinin, and 1% Triton X-100) in the presence of protease inhibitor and phosphatase inhibitor on ice. The lysate was centrifuged at 13,000 rpm for 10 min to obtain the supernatant. The protein concentrations were determined by the BCA protein assay kit (Boster, China). The lysed samples were separated by SDS-PAGE and then transferred to polyvinylidene fluoride (PVDF) membranes. After blocking with 5% skim milk for 2 h at room temperature, the membranes were incubated with specific primary antibodies at 4°C overnight. The PVDF membranes were then incubated with HRP-conjugated secondary antibodies for 2 h, and protein bands were captured via enhanced chemiluminescence. The gray intensity of the protein bands was analyzed by Image J software (NIH, Bethesda, MD).

### 2.12. Statistical Analysis

Data were expressed as mean ± SEM. Statistical significance was analyzed by one-way ANOVA in SPSS version 19.0. The Tukey test was used after ANOVA to perform multiple comparison analysis. Differences at *p* < 0.05 were considered statistically significant.

## 3. Results

### 3.1. XNJY Increased the Body Weight in a Rat Model of PSD

**Figure 1:**
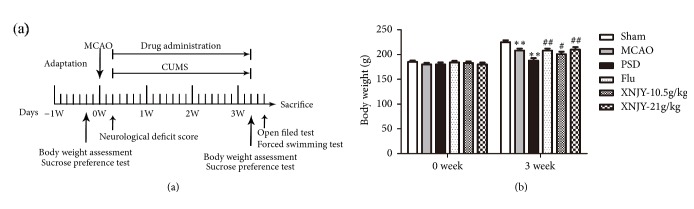
**XNJY increased the body weight in a rat model of PSD**. Time schedule of experimental procedures (a). Rat body weights in different groups during the experimental period (b). Data were expressed as mean ± SEM. Multiple comparison analysis was conducted by Tukey test after ANOVA. Differences at *p* < 0.05 were considered statistically significant. ^*∗∗*^*p* < 0.01, versus Sham group; ^#^*p* < 0.05, ^##^*p* < 0.01, versus PSD group.

Figure 1(a) shows the time schedule of the experimental procedures.  Figure 1(b) presents the body weights of the rats in different groups during the experimental period. After treatment of MCAO combined with CUMS, the body weight in the PSD group of rats decreased dramatically compared with that of the Sham group (*p* < 0.01). Compared with the PSD group rats, the rats given 10.5 g/kg XNJY (XNJY-10.5g/kg; *p* < 0.05), 21 g/kg XNJY (XNJY-21g/kg; *p* < 0.01), and fluoxetine hydrochloride (Flu; *p* < 0.01) presented significantly increased body weight.

### 3.2. XNJY Affected Depressive-Like Behaviors in a Rat Model of PSD

**Figure 2:**
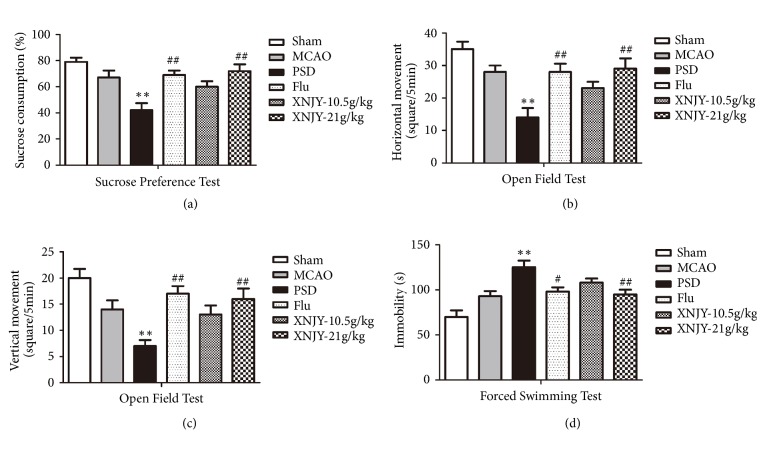
**XNJY ameliorated depressive-like behaviors in a rat model of PSD**. The depressive-like behaviors in rats were evaluated by sucrose preference test (a), open field test (b,c), and forced swimming test (d). Data were expressed as mean ± SEM. Multiple comparison analysis was conducted by Tukey test after ANOVA. Differences at *p* < 0.05 were considered statistically significant. ^*∗∗*^*p* < 0.01, versus Sham group; ^#^*p* < 0.05, ^##^*p* < 0.01, versus PSD group.

Depressive-like behaviors in rats were evaluated by sucrose preference test (Figure 2(a)), open field test (Figures 2(b) and 2(c)), and forced swimming test (Figure 2(d)). After 21 days of CUMS, we detected a significant decline in sucrose consumption, locomotor activity (including horizontal movement and vertical movement), and increased immobility time during forced swimming relative to those of the Sham group (*p* < 0.01). After Flu and XNJY-21g/kg administration, the sucrose consumption and locomotor activity of the rats were significantly higher than those of the PSD group rats. Meanwhile, Flu and XNJY-21g/kg treatment shortened the immobility time. Besides, XNJY-10.5 g/kg treatment induced a minimal response, and no significant difference with the PSD group was observed (*p* > 0.05).

### 3.3. XNJY Promoted Neuronal Survival in a Rat Model of PSD

**Figure 3:**
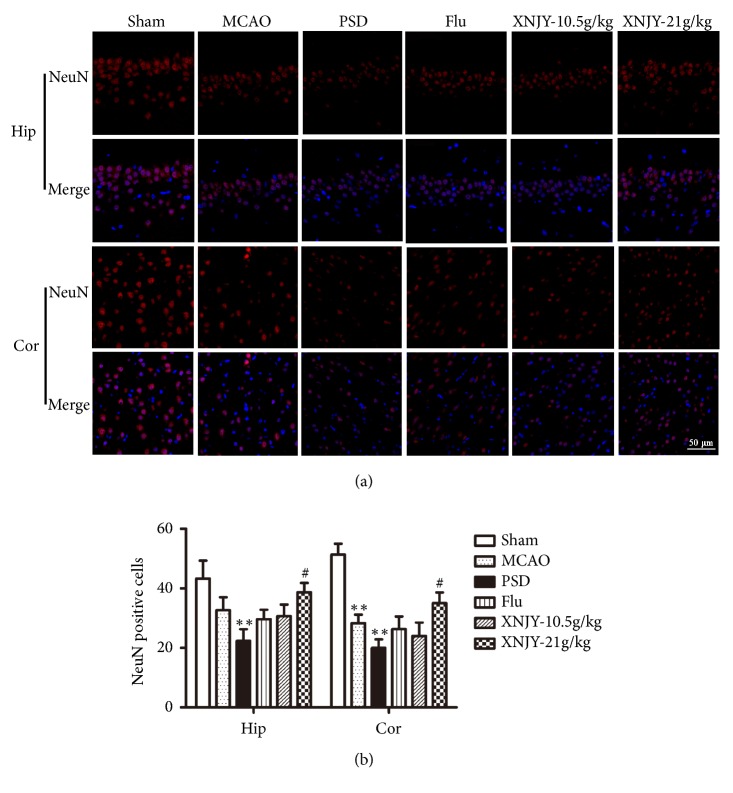
**XNJY promoted neuronal survival in a rat model of PSD**. Survival of neurons in the CA1 region of the hippocampus and prefrontal cortex (a). Graph shows the quantification of NeuN-positive cells (b). Data were expressed as mean ± SEM. Multiple comparison analysis was conducted by Tukey test after ANOVA. Differences at *p* < 0.05 were considered statistically significant. ^*∗∗*^*p* < 0.01, versus Sham group; ^#^*p* < 0.05, versus PSD group.

To investigate the survival of neurons in the CA1 region of the hippocampus and prefrontal cortex, we evaluated NeuN-positive cells (NeuN is a neuron specific marker) by immunofluorescence assay. As shown in Figures 3(a) and 3(b), the NeuN-positive cells of the rat model of PSD in the CA1 region and prefrontal cortex were severely reduced in number relative to those in the Sham group (*p* < 0.01). XNJY-21 g/kg treatment significantly increased the number of NeuN-positive cells in the CA1 region and prefrontal cortex relative to those in the PSD group rats (*p* < 0.01). Besides, the numbers of NeuN-positive cells only slightly changed in the Flu group and XNJY-10.5g/kg group relative to that in the PSD group (*p* > 0.05).

### 3.4. XNJY Protected Neurons against PSD-Induced Apoptosis

**Figure 4:**
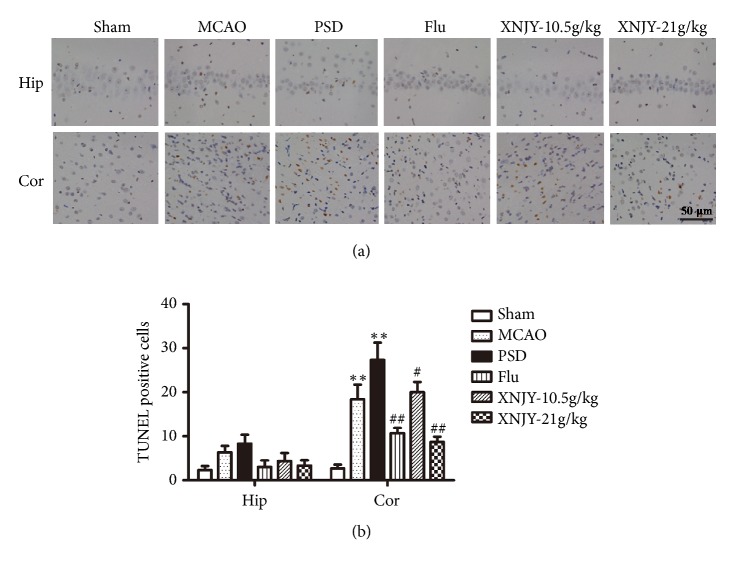
**XNJY protected neurons against PSD-induced apoptosis**. Apoptotic neurons in the CA1 region of hippocampus and prefrontal cortex (a). Graph shows the number of TUNEL-positive cells (b). Data were expressed as mean ± SEM. Multiple comparison analysis was conducted by Tukey test after ANOVA. Differences at *p* < 0.05 were considered statistically significant. ^*∗∗*^*p* < 0.01, versus Sham group; ^#^*p* < 0.05, ^##^*p* < 0.01, versus PSD group.

The neuronal apoptosis induced by PSD in the CA1 region of the hippocampus and prefrontal cortex was measured by TUNEL assay. Figures 4(a) and 4(b) show that the number of apoptotic cells in the PSD group significantly increased in the cortex relative to that in the Sham group (*p* < 0.01). However, no significant difference in apoptotic cell number was noted between the Sham group and PSD group in the CA1 region of the hippocampus (*p* > 0.05). After the Flu, XNJY-10.5 g/kg, and XNJY-21 g/kg treatments, the numbers of apoptotic cells in the cortex significantly declined.

### 3.5. XNJY Recovered the 5-HT, NE, and BDNF Contents in the Rat Models of PSD

**Figure 5:**
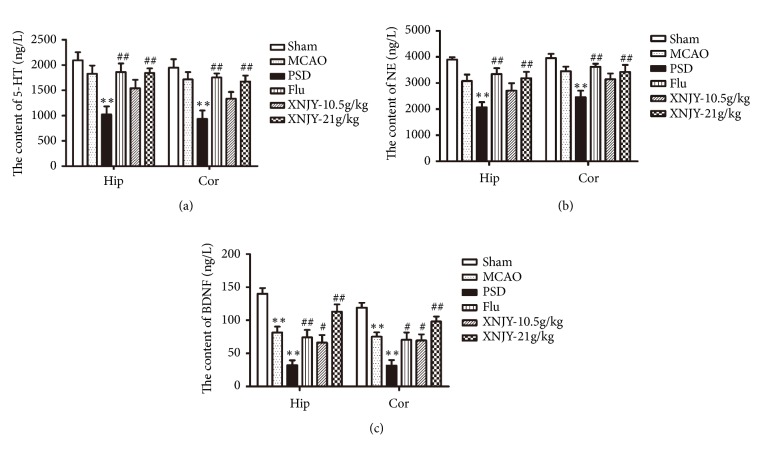
**XNJY recovered the 5-HT, NE, and BDNF contents in a rat model of PSD**. Contents of 5-HT (a), NE (b), and BDNF (c) in the hippocampus and cortex. Data were expressed as mean ± SEM. Multiple comparison analysis was conducted by Tukey test after ANOVA. Differences at *p* < 0.05 were considered statistically significant. ^*∗∗*^*p* < 0.01, versus Sham group; ^#^*p* < 0.05, ^##^*p* < 0.01, versus PSD group.

The levels of 5-HT, NE, and BDNF in the hippocampus and cortex are closely related to the pathogenesis of PSD. We detected the contents of 5-HT (Figure 5(a)), NE (Figure 5(b)), and BDNF (Figure 5(c)) in the hippocampus and cortex by using ELISA. Data showed that the contents of 5-HT and NE in the hippocampus and cortex were dramatically reduced in the PSD group than in the Sham group. By contrast, Flu and XNJY-21 g/kg treatment significantly recovered the levels of 5-HT and NE in the hippocampus and cortex (*p* < 0.01). XNJY-10.5 g/kg administration showed no effect on the levels of 5-HT and NE relative to those in the PSD group (*p* > 0.05). Moreover, the contents of BDNF in the hippocampus and cortex significantly decreased in the MCAO and PSD groups than in the Sham group (*p* < 0.01). Flu, XNJY-10.5 g/kg, and XNJY-21 g/kg treatments increased the level of BDNF in the hippocampus and cortex in varying degrees.

### 3.6. XNJY Regulated the BDNF/ERK/CREB Pathway in a Rat Model of PSD

**Figure 6:**
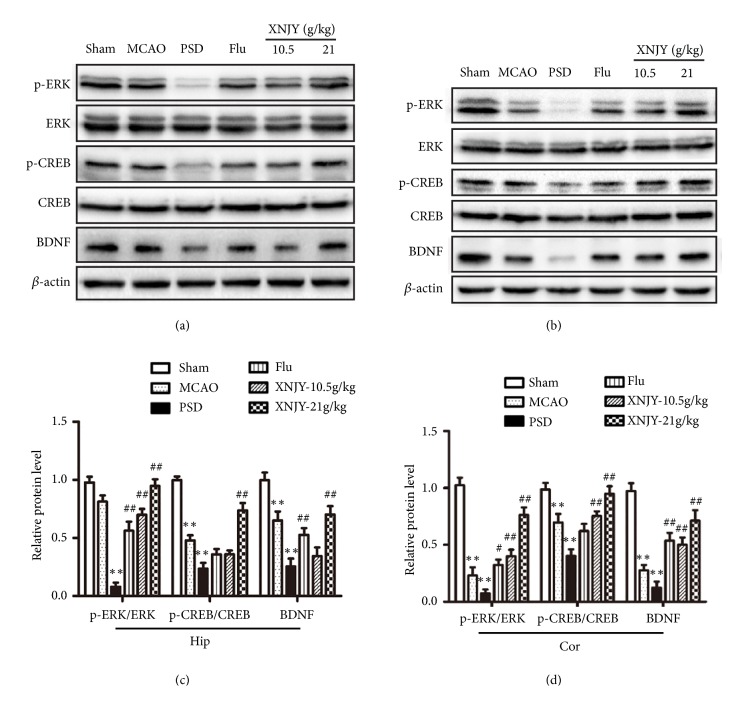
**XNJY regulated the BDNF/ERK/CREB pathway in a rat model of PSD**. Immunoblot levels of p-ERK, ERK, p-CREB, CREB, and BDNF in the hippocampus (a) and cortex (b). Graph shows the quantification of p-ERK/ERK, p-CREB/CREB, and BDNF protein levels in the hippocampus (c) and cortex (d). Data were expressed as mean ± SEM. Multiple comparison analysis was conducted by Tukey test after ANOVA. Differences at *p* < 0.05 were considered statistically significant. ^*∗∗*^*p* < 0.01, versus Sham group; ^#^*p* < 0.05, ^##^*p* < 0.01, versus PSD group.

To clarify the underlying mechanisms of XNJY decoction's antidepressant effects, we determined the protein levels of BDNF, ERK, p-ERK, CREB, and p-CREB in the hippocampus (Figures 6(a) and 6(c)) and cortex (Figures 6(b) and 6(d)). The PSD group demonstrated a decreased protein level for BDNF and diminished protein ratios of p-ERK/ERK and p-CREB/CREB in the hippocampus and cortex relative to those in the Sham group (*p* < 0.01). Flu treatment increased the protein levels of p-ERK/ERK and BDNF in the hippocampus and cortex (*p* < 0.01) but did not affect p-CREB/CREB (*P* > 0.05). XNJY-10.5 g/kg restored the ratio of p-ERK/ERK in the hippocampus and the levels of p-ERK/ERK, p-CREB/CREB, and BDNF in the cortex (*p* < 0.01). Moreover, XNJY-21 g/kg treatment significantly increased the levels of these indexes in the hippocampus and cortex (*p* < 0.01).

## 4. Discussion

In this study, we explored the antidepressant effects of XNJY decoction on a rat model of PSD and the underlying mechanisms. We observed that XNJY displayed antidepressant-like effects by increasing sucrose consumption, improving locomotor activity, and reducing the immobility time during forced swimming. Moreover, XNJY promoted neuronal survival in the cortex and hippocampus and protected neurons against apoptosis. XNJY also restored the levels of 5-HT, NE, and BDNF in the hippocampus and cortex and activated the BDNF/ERK/CREB signaling pathway.

PSD is a frequent complication of stroke that seriously affects the quality of life and rehabilitation of patients with stroke and even increases mortality [24, 25]. Currently, pharmacotherapy is still the main therapeutic approach for PSD despite the toxicity and side effects of the drugs and the drug withdrawal syndrome [26, 27]. Our results demonstrated that MCAO combined with CUMS could induce significant depressive-like behaviors, including body weight stagnation, decreased preference for sucrose water, decline in locomotor activity, and immobility time extension during forced swimming. XNJY could increase the body weight and improve the depressive-like behaviors in a rat model of PSD, and these effects were similar to those of Flu.

Depression following stroke is more common than in other physical illnesses with similar levels of physical disability; a direct biological link exists between stroke and depression [28, 29]. Neuronal loss and apoptosis are induced by ischemic or hemorrhagic stroke and can contribute to disorders of cognition, language, behavior, and emotion [30, 31]. Promoting neuronal survival, repair, and regeneration is valuable to prevent and treat stroke complications, such as PSD; this strategy has been considered a window for understanding antidepression management. In this study, the NeuN-positive cells of the PSD group rats significantly decreased in number in the hippocampus and cortex. XNJY promoted neuronal survival against the loss of NeuN-positive cells. Additionally, Flu and XNJY administration rescued the cortical neurons from apoptosis.

Depressive-like behaviors are associated with the reduced synthesis of monoamine transmitters, such as 5-HT and NE, in limbic areas of the frontal and temporal lobes [32]. Our study revealed that the hippocampal and cortical contents of 5-HT and NE in the PSD group were significantly lower than those in the Sham group. XNJY and Flu recovered the levels of 5-HT and NE in the hippocampus and cortex. Additionally, substantial evidence has revealed that the depression-associated neuronal atrophy, cell loss, and reduced tissue volume in human brains are partly mediated by a lack of sufficient neurotrophic support [33, 34]. BDNF, which belongs to the nerve growth factor family, plays an essential role in neuronal development, including growth, differentiation, and survival [35]. Patients with PSD and postmortem studies showed a decrease in BDNF protein and mRNA levels in the serum and hippocampus [36, 37]. Meanwhile, chronic antidepressant treatments can increase the BDNF level in vivo, and intrahippocampal and peripheral administration of BDNF produces antidepressant-like effects in rodent models with depression [38, 39]. In the present study, exposure to stress resulted in the decreased protein levels of BDNF in the hippocampus and cortex of PSD rats, whereas XNJY and Flu administration recovered such levels.

BDNF is an upstream regulator of the ERK cascade. The classic ERK1/2 cascade, as one of three major mitogen-activated protein kinase (MAPK) cascades, consists of the MAPK p44 ERK1 and p42 ERK2 [40]. The phosphorylation of ERK1/2 can activate the transcription factor CREB's gene expression, which has been proposed to be part of the response to various cellular stresses and operates by inducing the expression of most cAMP-regulated genes, including corticotropin-releasing factor and BDNF. The subsequent release of target proteins can promote neuronal survival, regeneration, and synaptic plasticity, thereby exerting antidepressant effects [41–43]. We observed that the protein levels of p-ERK/ERK, p-CREB/CREB, and BDNF in the hippocampus and cortex significantly decreased in the PSD group. Flu increased the levels of p-ERK/ERK and BDNF in the hippocampus and cortex but did not affect the ratio of p-CREB/CREB. However, XNJY significantly restored the levels of these indexes, which indicated that different from Flu, XNJY displayed antidepressant effects might through activating the BDNF/ERK/CREB pathway.

Our data demonstrated that XNJY exerted significant antidepressant effects by ameliorating depressive-like behaviors while promoting neuronal survival against apoptosis. Our study also found that XNJY activated the BDNF/ERK/CREB pathway and recovered the decreased levels of 5-HT, NE, and BDNF. These results suggested that XNJY may be a promising candidate for PSD treatment that acts by regulating the BDNF/ERK/CREB signaling pathway.

## Data Availability

The data used to support the findings of this study are available from the corresponding author upon request.

## Abbreviations

XNJY: Xingnao Jieyu
PSD: Poststroke depression
Flu: Fluoxetine
BDNF: Brain-derived neurotrophic factor
5-HT: Serotonin
NE: Norepinephrine
MCAO: Middle cerebral artery occlusions
CUMS: Chronic unpredictable mild stress
CREB: cAMP response element binding
TCM: Traditional Chinese medicine.

## References

[1] Babkair L. A. (2017). Risk Factors for Poststroke Depression: An Integrative Review. *Journal of Neuroscience Nursing*.

[2] Arseniou S., Arvaniti A., Samakouri M. (2011). Post-stroke depression: recognition and treatment interventions. *Psychiatriki*.

[3] Robinson R. G., Jorge R. E. (2016). Post-stroke depression: a review. *The American Journal of Psychiatry*.

[4] Kim J. S. (2016). Post-stroke mood and emotional disturbances:pharmacological therapy based on mechanisms. *Journal of Stroke*.

[5] Kallio M. E. (2014). Neuropsychiatric outcomes after stroke. *The Lancet Neurology*.

[6] Coupland C., Dhiman P., Morriss R., Arthur A., Barton G., Hippisley-Cox J. (2011). Antidepressant use and risk of adverse outcomes in older people: Population based cohort study. *BMJ*.

[7] Hackam D. G., Mrkobrada M. (2012). Selective serotonin reuptake inhibitors and brain hemorrhage: A meta-analysis. *Neurology*.

[8] Ferrari F., Villa R. F. (2017). The Neurobiology of Depression: an Integrated Overview from Biological Theories to Clinical Evidence. *Molecular Neurobiology*.

[9] Villa R. F., Ferrari F., Moretti A. (2018). Post-stroke depression: Mechanisms and pharmacological treatment. *Pharmacology & Therapeutics*.

[10] Espárrago Llorca G., Castilla-Guerra L., Fernández Moreno M., Ruiz Doblado S., Jiménez Hernández M. (2015). Post-stroke depression: an update. *Neurología (English Edition)*.

[11] Nestler E. J., Barrot M., DiLeone R. J., Eisch A. J., Gold S. J., Monteggia L. M. (2002). The neurobiology of depression. *Neuron*.

[12] Li J., Zhao Y.-D., Zeng J.-W., Chen X.-Y., Wang R.-D., Cheng S.-Y. (2014). Serum Brain-derived neurotrophic factor levels in post-stroke depression. *Journal of Affective Disorders*.

[13] Berretta A., Tzeng Y.-C., Clarkson A. N. (2014). Post-stroke recovery: the role of activity-dependent release of brain-derived neurotrophic factor. *Expert Review of Neurotherapeutics*.

[14] O'Keefe L. M., Doran S. J., Mwilambwe-Tshilobo L., Conti L. H., Venna V. R., McCullough L. D. (2014). Social isolation after stroke leads to depressive-like behavior and decreased BDNF levels in mice. *Behavioural Brain Research*.

[15] Jin H., Pei L., Li Y. (2017). Alleviative effects of fluoxetine on depressive-like behaviors by epigenetic regulation of BDNF gene transcription in mouse model of post-stroke depression. *Scientific Reports*.

[16] Kotlęga D., Peda B., Zembroń-Łacny A., Gołąb-Janowska M., Nowacki P. (2017). The role of brain-derived neurotrophic factor and its single nucleotide polymorphisms in stroke patients. *Neurologia i Neurochirurgia Polska*.

[17] Dwivedi Y., Rizavi H. S., Conley R. R., Roberts R. C., Tamminga C. A., Pandey G. N. (2003). Altered gene expression of brain-derived neurotrophic factor and receptor tyrosine kinase B in postmortem brain of suicide subjects. *Archives of General Psychiatry*.

[18] Vaynman S., Ying Z., Gomez-Pinilla F. (2004). Hippocampal BDNF mediates the efficacy of exercise on synaptic plasticity and cognition. *European Journal of Neuroscience*.

[19] Butler L., Pilkington K. (2013). Chinese Herbal Medicine and Depression: The Research Evidence. *Evidence-Based Complementary and Alternative Medicine*.

[20] Fan W., Wang Q., Yan Y. (2012). Influence of Xingnao Jieyu capsule on hippocampal and frontal lobe neuronal growth in a rat model of post-stroke depression. *Neural Regeneration Research*.

[21] Yan Y. M., Fan W. T., Liu L., Yang R., Yang W. J. (2013). The effects of Xingnao Jieyu capsules on post-stroke depression are similar to those of fluoxetine. *Neural Regeneration Research*.

[22] Longa E. Z., Weinstein P. R., Carlson S., Cummins R. (1989). Reversible middle cerebral artery occlusion without craniectomy in rats. *Stroke*.

[23] Grippo A. J., Beltz T. G., Weiss R. M., Johnson A. K. (2006). The effects of chronic fluoxetine treatment on chronic mild stress-induced cardiovascular changes and anhedonia. *Biological Psychiatry*.

[24] Schulz R., Beach S. R., Ives D. G., Martire L. M., Ariyo A. A., Kop W. J. (2000). Association between depression and mortality in older adults: the cardiovascular health study. *JAMA Internal Medicine*.

[25] Carod-Artal F. J. (2006). Post-stroke depression (II): its differential diagnosis, complications and treatment. *Revista de Neurología*.

[26] Paolucci S. (2008). Epidemiology and treatment of post-stroke depression. *Neuropsychiatric Disease and Treatment*.

[27] Xu X., Zou D., Shen L. (2016). Efficacy and feasibility of antidepressant treatment in patients with post-stroke depression. *Medicine*.

[28] Whyte E. M., Mulsant B. H. (2002). Post stroke depression: epidemiology, pathophysiology, and biological treatment. *Biological Psychiatry*.

[29] Hackett M. L., Pickles K. (2014). Part I: frequency of depression after stroke: an updated systematic review and meta-analysis of observational studies. *International Journal of Stroke*.

[30] Broughton B. R. S., Reutens D. C., Sobey C. G. (2009). Apoptotic mechanisms after cerebral ischemia. *Stroke*.

[31] Zhang Y., Zhao H., Fang Y., Wang S., Zhou H. (2017). The association between lesion location, sex and poststroke depression: Meta-analysis. *Brain and Behavior*.

[32] Terroni L. A., Amaro E., Iosifescu D. V. (2011). Stroke lesion in cortical neural circuits and post-stroke incidence of major depressive episode: a 4-month prospective study. *The World Journal of Biological Psychiatry*.

[33] Duclot F., Kabbaj M. (2015). Epigenetic mechanisms underlying the role of brain-derived neurotrophic factor in depression and response to antidepressants. *Journal of Experimental Biology*.

[34] De Azevedo Cardoso T., Mondin T. C., Wiener C. D. (2014). Neurotrophic factors, clinical features and gender differences in depression. *Neurochemical Research*.

[35] Schmidt H. D., Duman R. S. (2007). The role of neurotrophic factors in adult hippocampal neurogenesis, antidepressant treatments and animal models of depressive-like behavior. *Behavioural Pharmacology*.

[36] Dwivedi Y., Mondal A. C., Rizavi H. S., Conley R. R. (2005). Suicide brain is associated with decreased expression of neurotrophins. *Biological Psychiatry*.

[37] Lee B.-H., Kim Y.-K. (2010). BDNF mRNA expression of peripheral blood mononuclear cells was decreased in depressive patients who had or had not recently attempted suicide. *Journal of Affective Disorders*.

[38] Shirayama Y., Chen A. C.-H., Nakagawa S., Russell D. S., Duman R. S. (2002). Brain-derived neurotrophic factor produces antidepressant effects in behavioral models of depression. *The Journal of Neuroscience*.

[39] Schmidt H. D., Duman R. S. (2010). Peripheral BDNF produces antidepressant-like effects in cellular and behavioral models. *Neuropsychopharmacology*.

[40] Leal G., Comprido D., Duarte C. B. (2014). BDNF-induced local protein synthesis and synaptic plasticity. *Neuropharmacology*.

[41] Liu D., Wang Z., Gao Z. (2014). Effects of curcumin on learning and memory deficits, BDNF, and ERK protein expression in rats exposed to chronic unpredictable stress. *Behavioural Brain Research*.

[42] Chen Y.-H., Zhang R.-G., Xue F. (2015). Quetiapine and repetitive transcranial magnetic stimulation ameliorate depression-like behaviors and up-regulate the proliferation of hippocampal-derived neural stem cells in a rat model of depression: The involvement of the BDNF/ERK signal pathway. *Pharmacology Biochemistry & Behavior*.

[43] Arango-Lievano M., Lambert W. M., Bath K. G., Garabedian M. J., Chao M. V., Jeanneteau F. (2015). Neurotrophic-priming of glucocorticoid receptor signaling is essential for neuronal plasticity to stress and antidepressant treatment. *Proceedings of the National Acadamy of Sciences of the United States of America*.

